# ACO: Time to move from the description of different phenotypes to the treatable traits

**DOI:** 10.1371/journal.pone.0210915

**Published:** 2019-01-24

**Authors:** Nuria Toledo-Pons, Job F. M. van Boven, Miguel Román-Rodríguez, Noemí Pérez, Jose Luis Valera Felices, Joan B. Soriano, Borja G. Cosío

**Affiliations:** 1 CIBER Enfermedades Respiratorias (CIBERES), Instituto de Salud Carlos III, Madrid, Spain; 2 Department of Respiratory Medicine, Hospital Universitari Son Espases-IdISBa, Mallorca, Spain; 3 Department of General Practice & Elderly Care Medicine, Groningen Research Institute for Asthma and COPD (GRIAC), University Medical Centre Groningen, University of Groningen, Groningen, The Netherlands; 4 Primary Care respiratory research unit, Instituto de Investigación Sanitaria de las Islas Baleares (IdISBa), Mallorca, Spain; 5 Gabinete Técnico Servicios Centrales, Servicio de Salud de las Islas Baleares, Mallorca, Spain; 6 Hopital Universitario de la Princesa, Universidad Autónoma de Madrid, Madrid, Spain; 7 Consultor de Metodología e Investigación de SEPAR, Barcelona, Spain; National and Kapodistrian University of Athens, SWITZERLAND

## Abstract

**Background:**

Asthma-COPD overlap (ACO) is a term that encompasses patients with characteristics of two conditions, smoking asthmatics or COPD patients with asthma-like features such as high bronchodilator response or blood eosinophil count ≥300 cells/μL. The aim of this study was to compare the different phenotypes inside the ACO definition in a real-life population cohort.

**Methods:**

We analyzed patients from the MAJORICA cohort who had a diagnosis of asthma and/or COPD based on current guidelines, laboratory data in 2014 and follow-up until 2015. Prevalence of ACO according to the different criteria, demographic, clinical and functional characteristics, prescriptions and use of health resources data were compared between three groups.

**Results:**

We included 603 patients. Prevalence of smoking asthmatics was 14%, COPD patients with high bronchodilator response 1.5% and eosinophilic COPD patients 12%. Smoking asthmatics were younger and used more rescue inhalers, corticosteroids and health resources. Conversely, eosinophilic COPD patients were older than the other groups, often treated with corticosteroids and had lower use of health resources. Most of the COPD patients with high bronchodilator response were included in the eosinophilic COPD group.

**Conclusions:**

ACO includes two conditions (smoking asthmatics and eosinophilic COPD patients) with different medication requirement and prognosis that should not be pooled together. Use of ≥300 blood eosinophils/μL as a treatable trait should be recommended.

## Introduction

The GOLD-GINA consensus recommends combining three characteristics of asthma and three of chronic obstructive pulmonary disease (COPD) to make a diagnosis of overlap between asthma and COPD (ACO). The assumption that patients with ACO are all similar, irrespectively if the diagnosis comes from an asthma patient that smokes or from a COPD patient with clinical characteristics of asthma, has led to consider ACO as an homogeneous condition. However, recent studies have shown that ACO is actually an heterogeneous condition with clinical and inflammatory differences between smoking asthmatics and eosinophilic COPD [1, 2].

A recent publication proposed an algorithm to help clinicians to identify ACO among patients with chronic obstructive airway disease [3]. Firstly, it requires the diagnosis of COPD based on current guidelines [4]. Secondly, the diagnosis of ACO can be considered in three different scenarios: 1) if the patient has also a previous diagnosis of asthma, or 2) if the patient presents a high bronchodilator response (HBR, defined as a change of >400 ml and >15% in FEV_1_) and/or 3) a significant blood eosinophil count (≥300 cells/μL).

In view of these criteria, it is likely that an excessive importance is given to a HBR in order to diagnose ACO. GOLD-GINA consensus recommends the use of 15% and 400 mL as cut-off to define a HBR in ACO. However, there is evidence that up to 60% of patients with COPD may demonstrate reversibility [5, 6] and that this is highly variable over time [7]. No bronchodilator test cut-off value has demonstrated to predict different clinical outcomes, neither in asthma nor in COPD, and the prevalence of HBR in a population with chronic airflow obstruction or COPD is unknown. Thus, there is still no evidence that bronchodilator responsiveness characterizes a disorder such as ACO.

Another potential marker for ACO diagnosis is a Th2 signature, expressed by the blood eosinophil count as a surrogate marker of airway eosinophilia. Higher eosinophil counts have been associated with increased risk of exacerbations and therapeutic responsiveness to inhaled corticosteroids [8–11] in COPD patients. Nevertheless, whether asthmatics with chronic obstruction or COPD patients with high eosinophil count have similar clinical characteristics and response to inhaled corticosteroids (ICS) treatment has not been studied yet.

The hypothesis of the present study is that ACO is a heterogeneous entity due to the combination of two different conditions with different underlying mechanisms, prognosis and therapeutic needs. The aim of the present study was to compare the prevalence, clinical characteristics, lung function, laboratory data and prognosis of patients classified as ACO from the three different approaches recommended by the aforementioned guidelines: co-diagnosis of asthma, HBR or eosinophil blood count ≥300 cells/μL.

## Methods

### Study design and ethics

This study used a retrospective design with prospective follow-up from a health-related population database. The STROBE (Strengthening the Reporting of Observational Studies in Epidemiology) recommendations were followed [6]. Individuals registered for primary care in the Balearic Islands, Spain, during 2012 were included in the cohort that contains follow-up data until 2015.

The study protocol was assessed and approved by the Balearic Primary Care Research Committee. Because of the retrospective design and use of anonymized data, this study was exempted from ethics approval.

### Data source

Data were extracted from the Majorca Real-Life Investigation in COPD and Asthma (MAJORICA) cohort. The characteristics of this cohort have been described elsewhere [12]. Briefly, this cohort contains combined data from three different data sources: primary care database, hospital electronic charts and electronic prescription system in the Balearics, Spain. These three data sources cover almost all clinical characteristics of, and health-care use by, the residents of the Balearics islands (±1.1 million subjects). The MAJORICA cohort includes data from all patients ≥18 years of age with a primary care diagnosis of asthma and/or COPD in 2012 (N = 68,578). All demographics, clinical data, laboratory tests, lung function, as well as resource use and pharmacy dispense data for the period between 2012 and 2015 were extracted.

### Population

**Fig 1:**
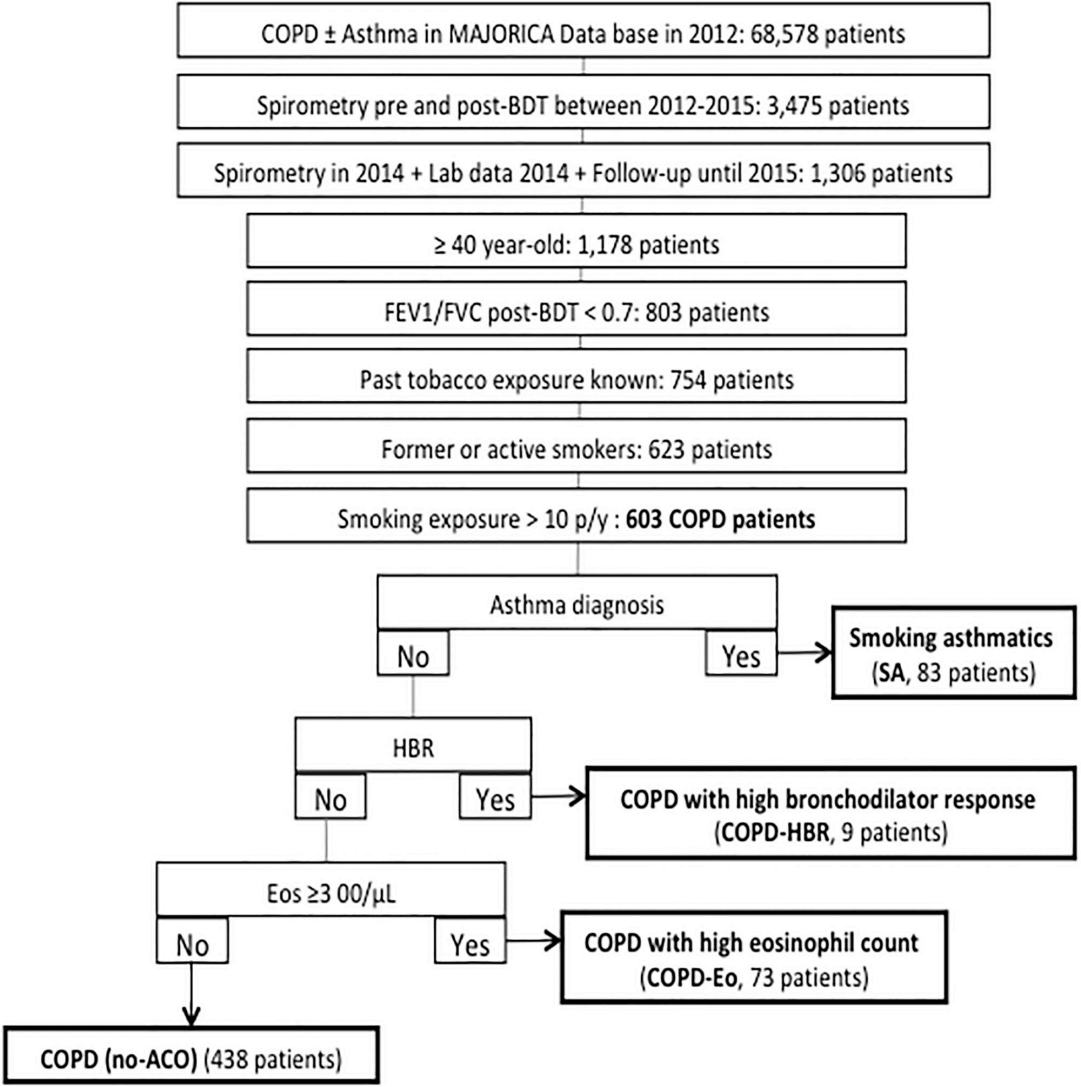
STROBE flow-chart. COPD: chronic obstructive pulmonary disease; BDT: bronchodilator test; FEV1: forced expiratory volume in the first second; FVC: forced vital capacity; p/y: pack-years; SA: smoking asthmatic; Eos: eosinophil; ACO: asthma-COPD overlap; HBR: high bronchodilator response.

We included patients from the MAJORICA cohort who had (1) ≥ 40 years of age; (2) smoking exposure > 10 pack-years; (3) spirometry confirmed post-bronchodilator airflow obstruction (FEV1/FVC < 0.7); (4) at least one eosinophil count in 2014; and (5) follow-up until 2015 (Fig 1).

### ACO definitions

Following a recently published algorithm aimed to identify ACO [3], we divided the population according the criteria fulfilled once the chronic airflow limitation and the tobacco exposure were demonstrated. Sequentially and mutually exclusive, firstly, we identify patients with a concomitant diagnosis of asthma and COPD (Smoking asthmatic, SA). Secondly, we distinguished patients with HBR, defined as bronchodilator response >400 ml and 15% in FEV1 (COPD-HBR). And thirdly, we discerned those patients with blood eosinophil count greater than 300 cells/μL (COPD-Eo). Thus, all patients who had received a physician confirmed diagnosis of both asthma (International Classification of Diseases, 9th revision [ICD-9] code: 493) and COPD (ICD-9 codes: 491, 492, and/or 496) in MAJORICA were identified as SA cases. Subsequently, patients coded as COPD with HBR were classified as COPD-HBR cases; and finally, other patients coded as COPD with a peripheral eosinophil count ≥ 300 eosinophils/μL in 2014 were classified as COPD-Eo cases. All other patients who did not meet any of these criteria were classified as COPD cases (Fig 1).

### Study size and data analysis

No formal sample size estimation was conducted because we were able to explore the entire population domain.

For quantitative and normally distributed variables results are expressed as mean ± standard deviation. If they are not normally distributed, results are presented with median and [interquartile range] or with median and (range) when numbers where too small. For categorical parameters, all groups are reported separately, using absolute number and percentage. ANOVA (or Kruskall-Wallis test) and unpaired t-tests (or Mann-Whitney U-tests) were used to compare normally (and abnormally) distributed quantitative variables. Chi-squared was used to compare categorical variables. Differences were considered statistically significant at 2-tailed p<0.05.

## Results

We included 603 patients who fulfilled all criteria, of which 165 were considered ACO according to the aforementioned diagnostic algorithm. ACO patients were younger, relatively more often female, showed less cardiovascular comorbidities and more osteoporosis and rhinitis, with more FEV1 reversibility, reduced rates of health resources use and more frequently treated with ICS and short-acting beta agonists (SABA) compared to COPD without ACO criteria (Table 1).

**Table 1:** Demographic and clinical characteristics of COPD and ACO populations.

	COPD (n = 438)	ACO (n = 165)	P-Value
Male	349 (79.7%)	108 (65.5%)	**<0.001**
Age, years	67.66 ± 9.12	63.38 ± 9.62	**<0.001**
Pack-years	16.12 ± 18.89	18.40 ± 21.50	0.230
**Comorbidities**			
Atrial Fibrillation	87 (19.9%)	14 (8.5%)	**0.001**
Anxiety, No. (%)	131 (29.9%)	58 (35.2%)	0.216
Osteoporosis	49 (11.2%)	29 (17.6%)	**0.037**
Allergic rhinitis	30 (6.8%)	21 (12.7%)	**0.021**
GERD, No. (%)	34 (7.8%)	16 (9.7%)	0.443
Nasal polyps, No. (%)	2 (0.5%)	3 (1.8%)	0.100
**Treatment**			
SABA	195 (44.5%)	91 (55.2%)	**0.020**
LAMA	318 (72.6%)	108 (65.5%)	0.086
LAMA-LABA	62 (14.2%)	15 (9.1%)	0.097
ICS	21 (4.8%)	8 (4.8%)	0.978
LABA-ICS	232 (53.0%)	107 (64.8%)	**0.009**
OCS	156 (35.6%)	46 (27.9%)	0.073
**Lung function**			
FVC postBD, liters	3.16 ± 0.91	3.28 ± 0.89	0.129
FVC postBD,% reference	85.50 ± 18.24	87.98 ± 16.83	0.117
FEV1 postBD, liters	1.65 ± 0.64	1.76 ± 0.61	**0.048**
FEV1 postBD,% reference	58.91 ± 19.34	61.85 ± 17.70	0.077
FEV1/FVC postBD	52.11 ± 12.70	53.61 ± 11.80	0.173
BDR			**<0.001**
•Negative	370 (84.5%)	111 (67.3%)	
•Positive (≥200ml and ≥12%)	68 (15.5%)	38 (23.0%)	
•Highly-positive (≥400ml and ≥15%)	0 (0%)	16 (9.7%)	
**Eosinophils count**			
Mean Eos	0.15 ± 0.07	0.33 ± 0.21	**<0.001**
Median Eos	0.14 ± 0.08	0.32 ± 0.21	**<0.001**
Maximum Eos	0.27 ± 0.19	0.47 ± 0.38	**<0.001**
**Use of health services**			
ED visits	1.74 ± 2.08	1.37 ± 1.95	**0.040**
Hosp all cause no.	1.14 ± 1.50	0.85 ± 1.50	**0.036**
Days of stay (all cause hosp)	9.60 ± 18.38	6.98 ± 19.28	0.133
Resp hosp no.	0.06 ± 0.28	0.10 ± 0.42	0.120
Days of stay (resp hosp)	0.40 ± 2.15	0.73 ± 3.55	0.167

P-Value (Chi-squared or T-student). **Bolded** text highlights variables with statistically significant differences (p≤0.05). COPD: chronic obstructive pulmonary disease; ACO: asthma-COPD overlap; SABA: short-acting beta agonists LABA: long-acting beta agonists; LAMA: long-acting muscarinic antagonists; ICS: inhaled corticosteroids; OCS: oral corticosteroids (at least one prescription during the study period); FEV1: forced expiratory volume in 1^st^ second; FVC: forced vital capacity; postBD: post-bronchodilator; BDR: bronchodilator response; Eos: eosinophils; ED: emergency department; Hosp: hospitalization; Resp hosp: respiratory hospitalization; No: number.

**Fig 2:**
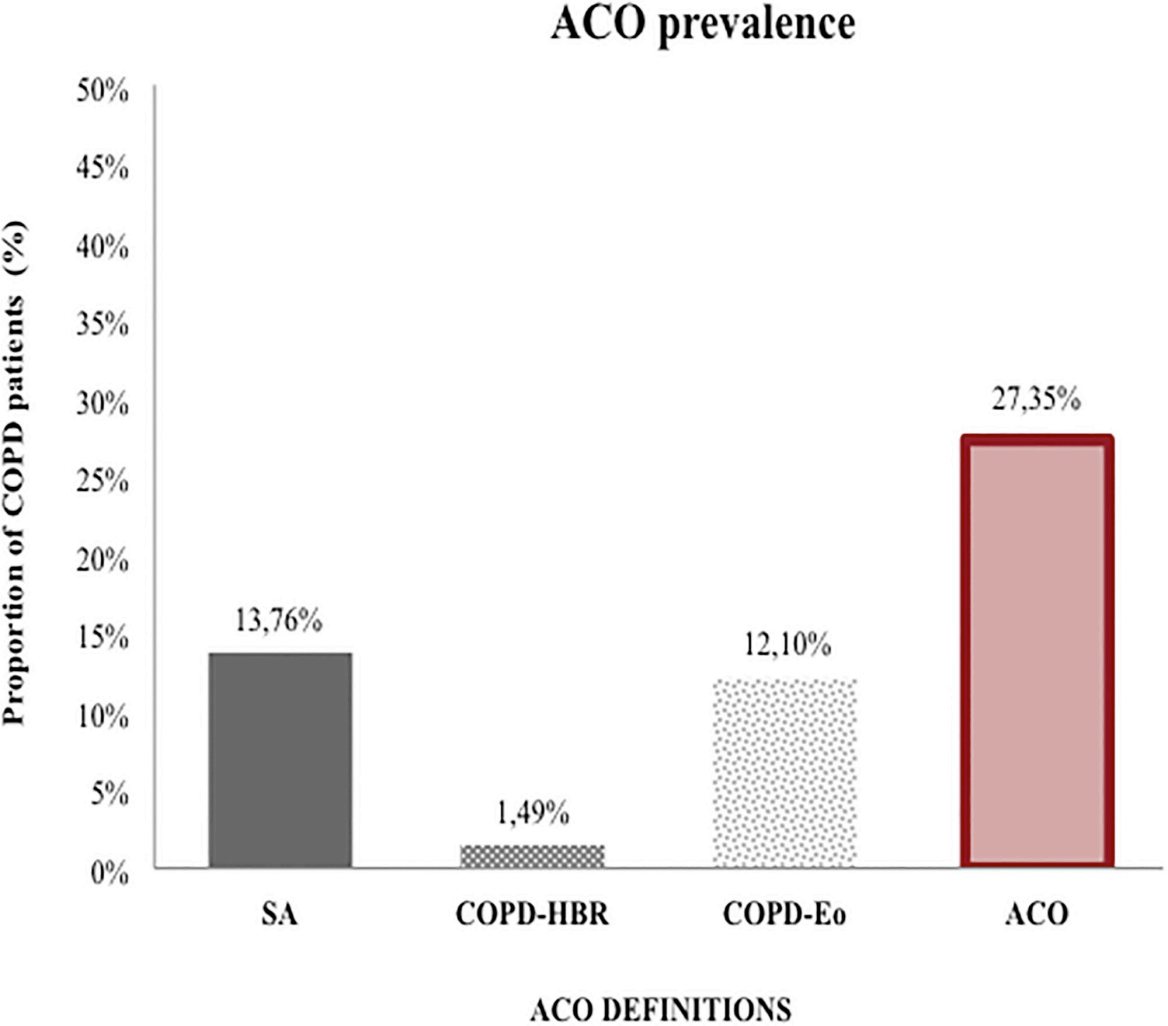
ACO prevalence. ACO: Asthma-COPD overlap; COPD: Chronic obstructive pulmonary disease; SA: smoking asthmatic; Eos: Eosinophil; HBR: High bronchodilator response.

Fig 2 shows the prevalence of ACO according to the different definitions used. The overall prevalence of ACO was 15 cases per 100,000 residents (≥ 18 years) of the Balearic Islands. SA prevalence was 13.8% (7.5 cases per 100,000 residents) and COPD-Eo prevalence was 12.1% (6,6 cases per 100,000 residents). These results contrast with the very low prevalence of the COPD-HBR group with only 1.5% (0.8 cases per 100,000 residents). The global prevalence of ACO after applying the algorithm was 27.4% within a well-characterized COPD population.

**Fig 3:**
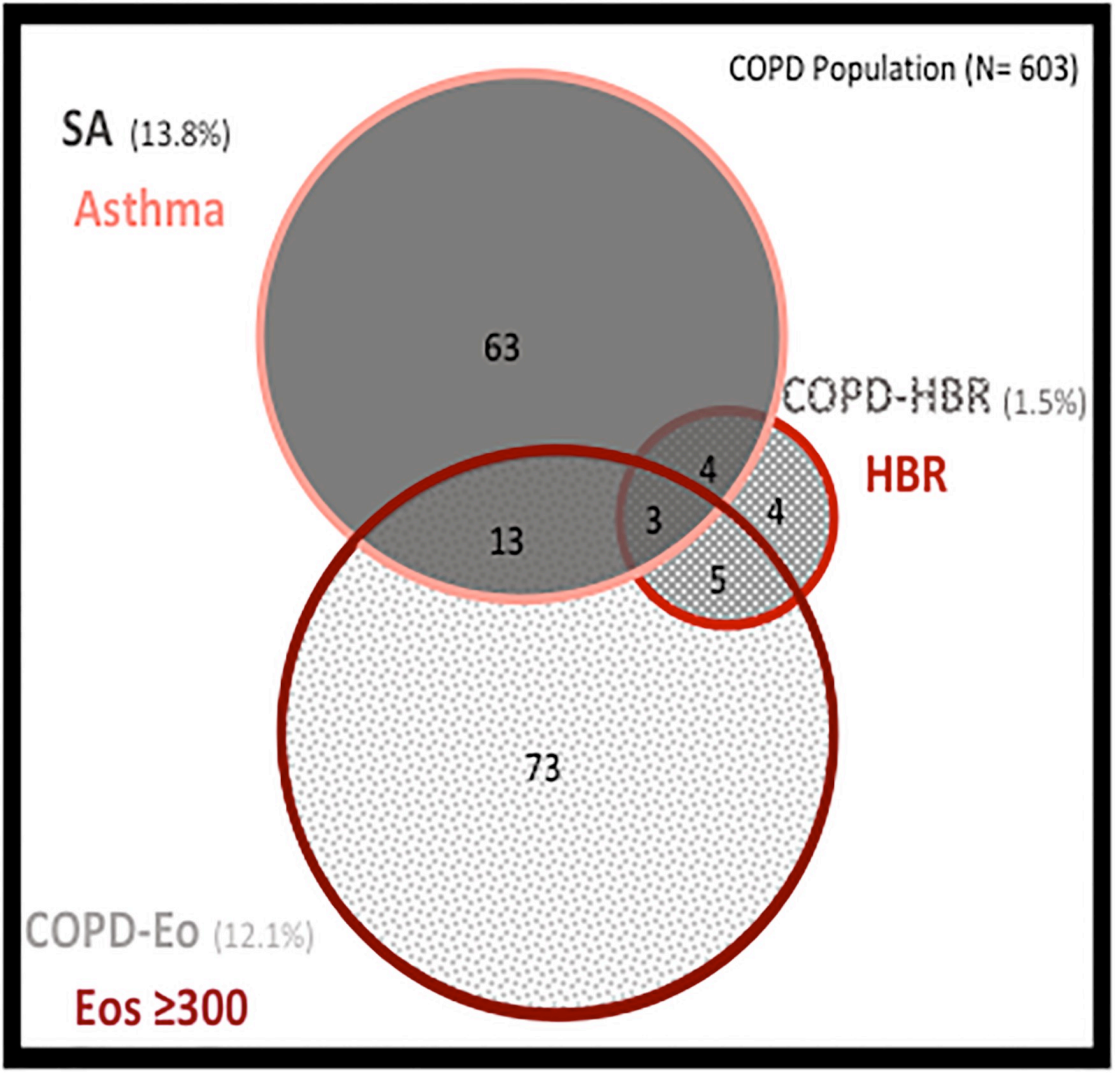
Venn diagram representing the overlap of the three ACO phenotypes. The square represents the entire COPD population. Patients who meet more than one definition of ACO are those who overlap with more than one circumference. COPD: Chronic obstructive pulmonary disease; Eos: Eosinophil; ACO: Asthma-COPD overlap; HBR: High bronchodilator response.

Although SA, COPD-HBR and COPD-Eo are diagnoses of exclusion, there can be some patients who present more than one defining characteristic at the same time (Fig 3). We observe that only a small proportion of patients with HBR are not included in the diagnoses of SA or COPD-Eo. On the contrary, despite there is an overlap between the characteristics of SA and COPD-Eo, these two populations present a significant and independent prevalence reflecting two differentiated populations.

### Comparison of the three ACO phenotypes

**Fig 4:**
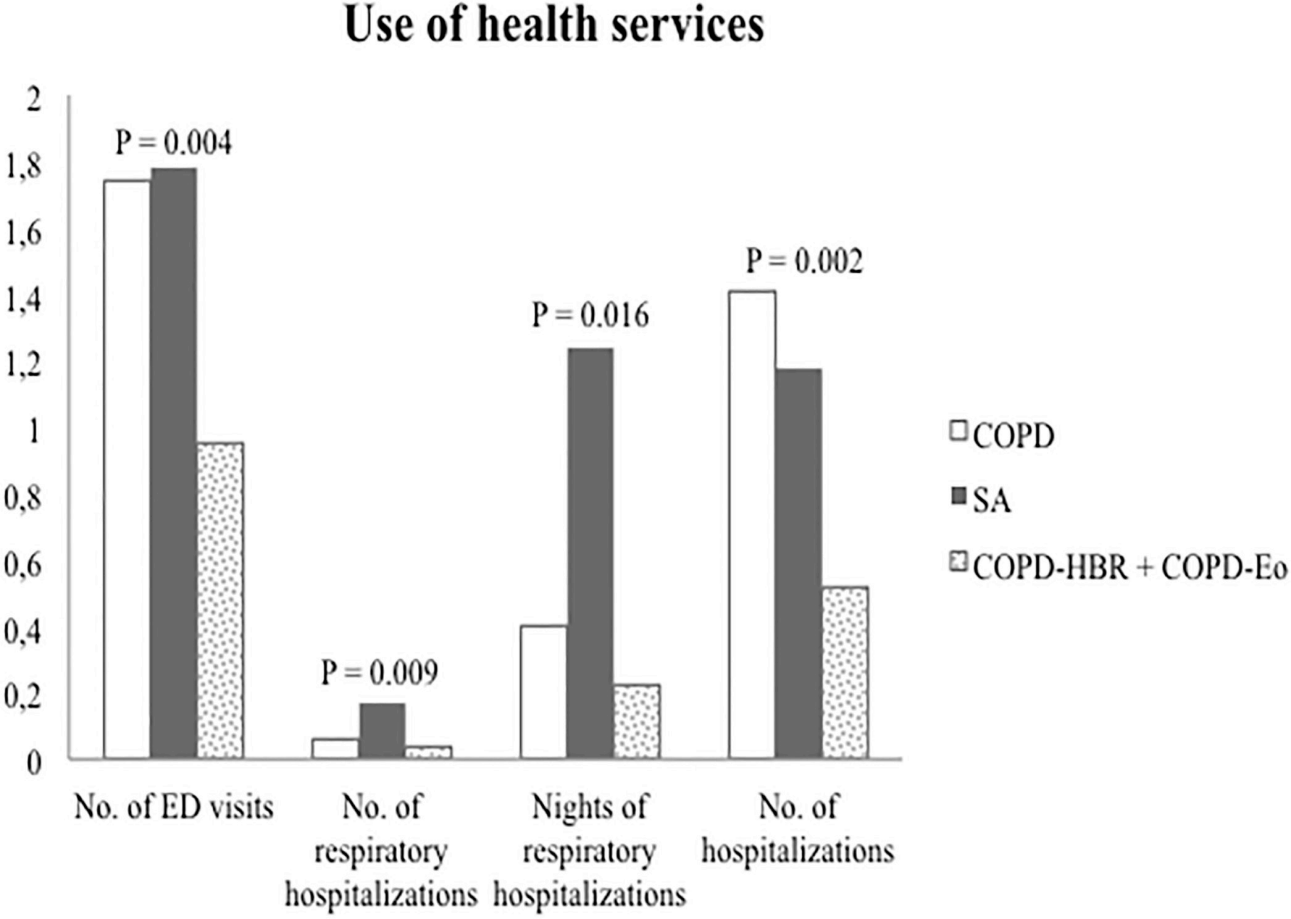
Use of health resources. COPD: Chronic obstructive pulmonary disease; ACO: Asthma-COPD overlap; No: number; ED: emergency department.

The demographic, clinical and functional characteristics of the three populations are shown in Table 2. SA patients were younger, relatively more often female and more frequently diagnosed of allergic rhinitis. This group, despite being younger, having similar cigarette smoke exposure and similar lung function used more SABA, ICS and oral corticosteroids (OCS) and made a higher use of health services compared to COPD-HBR and COPD-Eo (Fig 4). COPD-HBR patients were infrequent and shared almost all the characteristics with COPD-Eo patients. COPD-Eo patients were more frequently males, older than the other groups, often treated with corticosteroids, had higher eosinophil counts and lower rates of exacerbations.

**Table 2:** Demographic and clinical characteristics of the three ACO definitions.

	SA (n = 83)	COPD-HBR (n = 9)	COPD-Eo (n = 73)	P-Value
Male	47 (56.6%)	8 (88.9%)	53 (72.6%)^§^	**0.035**
Age, years	61.00 [53.00–67.00]	65.00 [58.50–68.50]*	66.00 [60.00–72.50]^§^	**0.002**
Pack-years	15.00 [4.00–21.00]	5.00 [2.00–39.00]	9.00 [3.00–26.50]	0.374
**Comorbidities**				
Atrial Fibrillation	9 (10.8%)	1 (11.1%)	4 (5.5%)	0.467
Anxiety, No. (%)	35 (42.2%)	3 (33.3%)	20 (27.4%)	0.155
Osteoporosis	17 (20.5%)	0 (0%)	12 (16.4%)	0.291
Allergic rhinitis	16 (19.3%)	0 (0%)	5 (6.8%)^§^	**0.034**
GERD, No. (%)	12 (14.5%)	0 (0%)	4 (5.5%)	0.100
Nasal polyps, No. (%)	3 (3.6%)	0 (0%)	0 (0%)	0.221
**Treatment**				
SABA	59 (71.1%)	4 (44.4%)	28 (38.4%)^§^	**<0.001**
LAMA	48 (57.8%)	8 (88.9%)	52 (71.2%)	0.067
LAMA-LABA	4 (4.8%)	1 (11.1%)	10 (13.7%)	0.153
ICS	7 (8.4%)	0 (0%)	1 (1.4%)	0.096
LABA-ICS	71 (85.5%)	3 (33.3%)*	33 (45.2%)^§^	**<0.001**
OCS	35 (42.2%)	1 (11.1%)	10 (13.7%)^§^	**<0.001**
**Lung function**				
FVC postBD, liters	3.23 [2.60–3.56]	3.77 [3.49–4.50]*	3.22 [2.72–3.81] ^♯^	**0.047**
FVC postBD,% reference	88.70 [77.80–97.60]	89.80 [75.05–98.35]	87.60 [74.85–99.80]	0.965
FEV1 postBD, liters	1.71 [1.31–2.10]	2.07 [1.43–2.75]	1.74 [1.29–2.10]	0.249
FEV1 postBD,% reference	59.10 [47.80–74.70]	67.70 [50.70–74.60]	62.80 [49.35–76.10]	0.716
FEV1/FVC postBD	56.40 [43.80–63.10]	52.90 [46.60–61.70]	56.60 [46.60–63.90]	0.958
BDR				**<0.001**
•Negative	54 (65.1%)	0 (0%)*	57 (78.1%)^§^	**<0.001**
•Positive (≥200 ml and ≥12%)	22 (26.5%)	0 (0%)*	16 (21.9%)	**<0.001**
•Highly-positive (≥400ml and ≥15%)	7 (8.4%)	9 (100%)*	0 (0%)^§^	**<0.001**
**Eosinophils count**				
Mean Eos	0.18 [0.10–0.29]	0.31 [0.23–0.43]*	0.37 [0.34–0.49]^§^	**<0.001**
Median Eos	0.15 [0.09–0.30]	0.29 [0.24–0.41]*	0.38 [0.33–0.48]^§^	**<0.001**
Maximum Eos	0.27 [0.17–0.40]	0.43 [0.27–0.75]*	0.49 [0.41–0.69]^§^	**<0.001**
**Use of health services**				
ED visits	1.00 (0–12)	1.00 (0–7)	0 (0–6)^§^	**0.044**
Hosp all cause no.	1.00 (0–13)	1.00 (0–4)	0 (0–3)^§^	**0.020**
Days of stay (all cause hosp)	2.00 (0–205)	5.00 (0–28)	0 (0–37)^§^	**0.022**
Resp hosp no.	0 (0–3)	0 (0–0)	0 (0–2)^§^	0.093
Days of stay (resp hosp)	0 (0–35)	0 (0–0)	0 (0–9)	0.095

P-Value (Chi-squared or Kruskal-Wallis). **Bolded** text highlights variables with statistically significant differences (p≤0.05).

*P-value <0.05 between SA and COPD-HBR;

^§^P-value <0.05 between SA and COPD-Eo;

^♯^P-value < 0.05 between COPD-HBR and COPD-Eo. COPD: chronic obstructive pulmonary disease; ACO: asthma-COPD overlap; HBR: high bronchodilator response; Eo: eosinophil; SABA: short-acting beta agonists LABA: long-acting beta agonists; LAMA: long-acting muscarinic antagonists; ICS: inhaled corticosteroids; OCS: oral corticosteroids (at least one prescription during the study period); FEV1: forced expiratory volume in 1^st^ second; FVC: forced vital capacity; postBD: post-bronchodilator; BDR: bronchodilator response; ED: emergency department; Hosp: hospitalization; Resp hosp: respiratory hospitalization; No: number.

### Differential characteristics of smoking asthmatics (SA) and COPD with asthma features (COPD-HBR+COPD-Eo) populations

As previously mentioned, SA patients were younger, relatively more often women and they presented more asthma-related comorbidities (allergic rhinitis and GERD, Table 3).

**Table 3:** Demographic and clinical characteristics of smoker asthmatic (SA) and COPD with asthma features (COPD-HBR+COPD-Eo) populations.

	SA (n = 83)	COPD-HBR+COPD-Eo (n = 82)	P-Value
Male	47 (56.6%)	61 (74.4%)	**0.016**
Age, years	60.98 ± 9.67	65.82 ± 8.99	**0.001**
Pack-years	20.29 ± 23.61	16.5 ± 19.08	0.259
**Comorbidities**			
Atrial Fibrillation	9 (10.8%)	5 (6.1%)	0.274
Anxiety, No. (%)	35 (42.2%)	23 (28%)	0.058
Osteoporosis	17 (20.5%)	12 (14.6%)	0.324
Allergic rhinitis	16 (19.3%)	5 (6.1%)	**0.011**
GERD, No. (%)	12 (14.5%)	4 (4.9%)	**0.038**
Nasal polyps, No. (%)	3 (3.6%)	0 (0%)	0.082
**Treatment**			
SABA	59 (71.1%)	32 (39.0%)	**<0.001**
LAMA	48 (57.8%)	60 (73.2%)	**0.038**
LAMA-LABA	4 (4.8%)	11 (13.4%)	0.055
ICS	7 (8.4%)	1 (1.2%)	**0.031**
LABA-ICS	71 (85.5%)	36 (43.9%)	**<0.001**
OCS	35 (42.2%)	11 (13.4%)	**<0.001**
**Lung function**			
FVC postBD, liters	3.21 ± 0.8	3.36 ± 0.98	0.279
FVC postBD,% reference	87.44 ± 14.52	88.52 ± 18.95	0.683
FEV1 postBD, liters	1.71 ± 0.59	1.81 ± 0.62	0.253
FEV1 postBD,% reference	60.63 ± 17.96	63.08 ± 17.47	0.375
FEV1/FVC postBD	53.08 ± 12.8	54.15 ± 10.74	0.562
BDR			0.529
•Negative	54 (65.1%)	57 (69.5%)	0.512
•Positive (≥200ml and ≥12%)	22 (26.5%)	16 (19.5%)	0.542
•Highly-positive (≥400ml and ≥15%)	7 (8.4%)	9 (11.0%)	0.581
**Eosinophils count**			
Mean Eos	0.23 ± 0.22	0.43 ± 0.16	**<0.001**
Median Eos	0.22 ± 0.22	0.41 ± 0.15	**<0.001**
Maximum Eos	0.33 ± 0.26	0.61 ± 0.42	**<0.001**
**Use of health services**			
ED visits	1.78 ± 2.29	0.95 ± 1.43	**0.006**
Hosp all cause no.	1.18 ± 1.89	0.52 ± 0.84	**0.004**
Days of stay (all cause hosp)	10.33 ± 25.92	3.59 ± 7.02	**0.024**
Resp hosp no.	0.17 ± 0.54	0.04 ± 0.25	**0.044**
Days of stay (resp hosp)	1.24 ± 4.77	0.22 ± 1.4	0.064

P-Value (Chi-squared or T-student). **Bolded** text highlights variables with statistically significant differences (p≤0.05). COPD: chronic obstructive pulmonary disease; ACO: asthma-COPD overlap; HBR: high bronchodilator response; Eo: eosinophil; SABA: short-acting beta agonists LABA: long-acting beta agonists; LAMA: long-acting muscarinic antagonists; ICS: inhaled corticosteroids; OCS: oral corticosteroids (at least one prescription during the study period); FEV1: forced expiratory volume in 1^st^ second; FVC: forced vital capacity; postBD: post-bronchodilator; BDR: bronchodilator response; ED: emergency department; Hosp: hospitalization; Resp hosp: respiratory hospitalization; No: number.

SA patients showed greater use of SABA and corticosteroids (oral and inhaled). On the contrary, the COPD-HBR+COPD-Eo group used more long-acting muscarinic antagonists (LAMA). COPD-HBR+COPD-Eo presented a higher eosinophil count. Despite no differences in lung function, the SA patients showed higher number of hospitalizations.

### Differential characteristics between COPD (non-ACO) vs COPD with asthma features (COPD-HBR+COPD-Eo) populations

When excluding the previous diagnosis of asthma, COPD patients with eosinophil counts≥300 (COPD-Eo) or HBR (COPD-HBR) were similar to COPD patients without these criteria in terms of age, smoking history and baseline lung function. However, non-ACO COPD patients presented more exacerbations, as defined by a higher use of OCS and health services compared to COPD-HBR+COPD-Eo patients (S1 Table).

### Comparison between COPD (non-ACO) vs smoking asthmatic (SA) populations

SA patients were younger and relatively more frequently females, presented more asthma-related comorbidities, reversibility and a higher use of SABA and ICS. The number of respiratory hospitalizations and hospital nights were increased in the SA group (S2 Table).

## Discussion

In this study, we have validated a new proposed algorithm to differentiate a specific phenotype of COPD in a population cohort. Using the aforementioned algorithm we identified that 27.4% of all COPD patients fulfilled the definition of ACO, and these patients were more frequently treated with ICS and showed a better prognosis in terms of healthcare utilization (emergency visits and all-cause hospitalizations). Moreover, we have addressed the heterogeneity of this group of patients classified under the umbrella of ACO, and differentiate eosinophilic COPD from those patients with COPD with a previous diagnosis of asthma as different entities with different clinical characteristics and prognosis in terms of hospital admissions and visits to emergency department. This findings remark a change of perspective when approaching this topic. Maybe, is time to abandon the search of phenotypes and to start finding treatable traits and specific biomarkers, to guide clinicians to customize the treatment of the patients.

### Previous studies

The need for a definition to adequately identify those patients who have features of COPD and asthma has been a reality and a topic of debate in recent years [13, 14]. However, the first studies that were conducted to study the overlap of Asthma and COPD, already noticed the heterogeneity of this entity [15]. Therefore, diagnostic approaches based on the study of inflammatory patterns in patients with COPD, asthma and patients with ACO have been proposed [16].

Some authors have used blood eosinophils and HBR as criteria to identify ACO patients [17, 18]. Sin *et al* proposed a consensus definition for ACO with four major and three minor criteria [18]. HBR was proposed as a major criterion if no history of asthma before 40 years was documented, and blood eosinophil count was considered a minor criterion.

The major differences in the definition of ACO used across the studies have led to incongruent data regarding its prevalence. Despite this, when the diagnosis of ACO is made in adults with COPD and previous diagnosis of asthma (SA), the prevalence varies between 13% and 18% [12, 19, 20]. In a recent study that applied the same proposed algorithm in a population of patients with chronic airflow limitation the prevalence was 29.8% [21]. These data would agree with the SA prevalence (13.8%) and with the total prevalence of ACO (27.4%) found in this cohort.

Regarding the evidence that currently exists on ACO prognosis, contradictory data are found, probably also related to different definitions used. When comparing COPD and ACO patients (defined by previous asthma diagnosis and/or asthma-like features), ACO patients showed better prognosis in terms of survival [20], lung function and exacerbations, even after 10-years of follow-up [22]. These findings are consistent with the Casanova *et al* results, in which the impact of persistent blood eosinophilia on exacerbations and survival was studied in COPD patients and smoking controls [23]. Despite not finding differences in the rate of exacerbations, mortality was lower in patients with blood eosinophil count ≥ 300 cells/μL compared to those with lower values [23]. On the contrary, Turato *et al* found no relationship between blood eosinophil levels and the rate of exacerbations or mortality in both, COPD patients or smoking controls. Of note, 61% of COPD patients were receiving ICS despite being predominantly of mild to moderate severity, which likely might contribute to equalize differences with non-Eosinophilic COPD [24].

The heterogeneity within ACO has already been explored [1, 25]. Pérez-de-Llano *et al* studied clinical and inflammatory profile differences between smoking asthmatics with airflow obstruction (SA) and eosinophilic inflammation COPD patients (COPD-Eo) [1]. They found that smoking asthmatics were more often females and had more atopic features. However, Th2-related biomarkers (periostin and FeNO) were higher in eosinophilic COPD patients. This heterogeneity could explain why other authors describe ACO patients as subjects with worse prognosis in relation to COPD patients. Hardin and colleagues compared subjects with both COPD and asthma (SA) with COPD alone [19]. They found ACO patients had worse health-related quality of life and experienced more exacerbations despite younger age. These results are consistent with our results where the SA group showed higher number of hospitalizations compared to the COPD-HBR+COPD-Eo group and COPD (non-ACO) group.

### Interpretation of results

The global prevalence of ACO, 27.4% is not negligible. Therefore, the effort of the scientific community to try to understand this condition is reasonable. Nevertheless, the criteria of HBR (COPD-HBR) had a very low prevalence (1.5%) and more than 50% of these patients had blood eosinophil levels higher than 300 cells/μL. These factors suggest that this entity is not clinically relevant. Most of these patients would be included in COPD-Eo group.

We have shown that ACO is an umbrella term that includes two conditions with different clinical characteristics and prognosis. The common belief that ACO confers poorer prognosis [19] comes from the patients that we have considered as SA, which are smoking asthmatics. However, ACO patients with HBR or mainly high eosinophil counts without asthma (COPD-HBR+COPD-Eo) present better prognosis than COPD without ACO and COPD with asthma (SA). A possible explanation about the better prognosis of COPD-HBR+COPD-Eo could be the use of inhaled corticosteroids, which is over 50% in these patients.

On the other hand, just 20.5% of SA patients present an eosinophil count ≥300 cells/μL. Therefore, a proportion of this population is likely to have a neutrophilic asthma phenotype, which is currently known to respond poorly to corticosteroids. This characteristic would justify the poorer prognosis of the SA group despite being younger with no differences in lung function compared with the COPD non-ACO and COPD-HBR+COPD-Eo groups.

### Clinical implications

One objective of defining new phenotypes in COPD is to find the most suitable treatment for each one. The GOLD-GINA consensus recommends the use of ICS in patients with ACO.

Therefore, regarding to ICS treatment recommendation, the proposed algorithm is valid mainly for the COPD-Eo group, since the COPD-HBR is not clinically relevant and the SA may benefit from other future biological therapies.

### Limitations

Our study has several limitations. First, this is a retrospective cohort study and therefore we were not able to address how COPD and asthma interact to modify the prognosis. Second, there is no gold standard for the diagnosis of asthma in a COPD population. Third, the cut-off eosinophil point to define COPD-Eo is arbitrary; a threshold of 300 eosinophils/μL was chosen following previous studies [3, 21, 23]. Fourth, our patients were recruited from public health services with universal health care and they were receiving treatment for COPD according to clinical practice; this could affect the results of the clinical outcomes like blood eosinophils or exacerbations. Results are not directly generalizable to patients coming from asthma clinics. Finally, 1-year follow-up might be too short to register COPD exacerbations.

## Conclusions

ACO is a prevalent and heterogeneous disorder that generates confusion because includes patients with different pathophysiology and prognosis, namely smoking asthmatics and eosinophilic COPD. This important heterogeneity leads us to think that probably using the term ACO to define all these patients can be confusing. From the practical point of view, the eosinophilic COPD (COPD-Eo group) identifies those COPD patients that would benefit the most from ICS. Moreover, the role of HBR is negligible and should not be used to identify this condition. We propose to abandon this term, and modify treatment of COPD by using a treatable trait such as the Th2 signature, using the blood eosinophil count as a marker. We should investigate now what is the best eosinophil cut-off point to predict the effective response to ICS.

## Supporting information

S1 Table Demographic and clinical characteristics of COPD (non-ACO) and COPD with asthma features (COPD-HBR + COPD-Eo) populations. P-Value (Chi-squared or T-student). **Bolded** text highlights variables with statistically significant differences (p≤0.05). COPD: chronic obstructive pulmonary disease; ACO: asthma-COPD overlap; SABA: short-acting beta agonists LABA: long-acting beta agonists; LAMA: long-acting muscarinic antagonists; ICS: inhaled corticosteroids; OCS: oral corticosteroids (at least one prescription during the study period); FEV1: forced expiratory volume in 1^st^ second; FVC: forced vital capacity; postBD: post-bronchodilator; BDR: bronchodilator response; Eos: eosinophils; ED: emergency department; Hosp: hospitalization; Resp hosp: respiratory hospitalization; No: number.
(DOCX)

S2 Table Demographic and clinical characteristics of COPD (no-ACO) and smoking asthmatic (SA) populations. P-Value (Chi-squared or T-student). **Bolded** text highlights variables with statistically significant differences (p≤0.05). vCOPD: chronic obstructive pulmonary disease; ACO: asthma-COPD overlap; SABA: short-acting beta agonists LABA: long-acting beta agonists; LAMA: long-acting muscarinic antagonists; ICS: inhaled corticosteroids; OCS: oral corticosteroids (at least one prescription during the study period); FEV1: forced expiratory volume in 1^st^ second; FVC: forced vital capacity; postBD: post-bronchodilator; BDR: bronchodilator response; Eos: eosinophils; ED: emergency department; Hosp: hospitalization; Resp hosp: respiratory hospitalization; No: number.
(DOCX)
