# Supplementary material for: ACO: Time to move from the description of different phenotypes to the treatable traits
Source: PLoS One. 2019 Jan 24;14(1):e0210915. doi: 10.1371/journal.pone.0210915 (PMC6345463; doi:10.1371/journal.pone.0210915)
Supplement: S1 Table — P-Value (Chi-squared or T-student). Bolded text highlights variables with statistically significant differences (p≤0.05). COPD: chronic obstructive pulmonary disease; ACO: asthma-COPD overlap; SABA: short-acting beta agonists LABA: long-acting beta agonists; LAMA: long-acting muscarinic antagonists; ICS: inhaled corticosteroids; OCS: oral corticosteroids (at least one prescription during the study period); FEV1: forced expiratory volume in 1st second; FVC: forced vital capacity; postBD: post-bronchodilator; BDR: bronchodilator response; Eos: eosinophils; ED: emergency department; Hosp: hospitalization; Resp hosp: respiratory hospitalization; No: number. (DOCX) [file pone.0210915.s001.docx]

**S1 Table. Demographic and clinical characteristics of COPD (non-ACO) and COPD with asthma features (COPD-HBR + COPD-Eo) populations.**

|  | **COPD (n=438)** | **COPD-HBR+COPD-Eo (n=82)** | **P-Value** |
| --- | --- | --- | --- |
| Male | 349 (79.7%) | 61 (74.4%) | 0.282 |
| Age, years | 67.66 ± 9.12 | 65.82 ± 8.99 | 0.091 |
| Pack years | 16.12 ± 18.89 | 16.5 ± 19.08 | 0.868 |
| **Comorbidities** |  |  |  |
| Atrial fibrillation | 87 (19.9%) | 5 (6.1%) | **0.003** |
| Anxiety, No. (%) | 131 (29.9%) | 23 (28%) | 0.735 |
| Osteoporosis, No. (%) | 49 (11.2%) | 12 (14.6%) | 0.373 |
| Allergic rhinitis, No. (%) | 30 (6.8%) | 5 (6.1%) | 0.803 |
| GERD, No. (%) | 34 (7.8%) | 4 (4.9%) | 0.357 |
| Nasal polyps, No. (%) | 2 (0.5%) | 0 (0%) | 0.580 |
| **Treatment** |  |  |  |
| SABA | 195 (44.5%) | 32 (39.0%) | 0.357 |
| LAMA | 318 (72.6%) | 60 (73.2%) | 0.916 |
| LAMA-LABA | 62 (14.2%) | 11 (13.4%) | 0.859 |
| ICS | 21 (4.8%) | 1 (1.2%) | 0.140 |
| LABA-ICS | 232 (53.0%) | 36 (43.9%) | 0.132 |
| OCS | 156 (35.6%) | 11 (13.4%) | **<0.001** |
| **Lung function** |  |  |  |
| FVC postBD, liters | 3.16 ± 0.91 | 3.36 ± 0.98 | 0.088 |
| FVC postBD, % reference | 85.50 ± 18.24 | 88.52 ± 18.95 | 0.186 |
| FEV1 postBD, liters | 1.65 ± 0.64 | 1.81 ± 0.62 | **0.029** |
| FEV1 postBD, % reference | 58.91 ± 19.34 | 63.08 ± 17.47 | 0.053 |
| FEV1/FVC postBD | 52.11 ± 12.70 | 54.15 ± 10.74 | 0.172 |
| BDR |  |  | **<0.001** |
| - Negative | 370 (84.5%) | 57 (69.5%) | **<0.001** |
| - Positive (≥200ml and ≥12%) | 68 (15.5%) | 16 (19.5%) | **<0.001** |
| - Highly-positive (≥400ml and ≥15%) | 0 (0%) | 9 (11.0%) | **<0.001** |
| **Eosinophils count** |  |  |  |
| Mean Eos | 0.15 ± 0.07 | 0.43 ± 0.16 | **<0.001** |
| Median Eos | 0.14 ± 0.08 | 0.41 ± 0.15 | **<0.001** |
| Maximum Eos | 0.27 ± 0.19 | 0.61 ± 0.42 | **<0.001** |
| **Use of health services** |  |  |  |
| ED visits | 1.74 ± 2.08 | 0.95 ± 1.43 | **0.001** |
| Hosp all cause no. | 1.14 ± 1.50 | 0.52 ± 0.84 | **<0.001** |
| Days of stay (all cause hosp) | 9.60 ± 18.38 | 3.59 ± 7.02 | **<0.001** |
| Resp hosp no. | 0.06 ± 0.28 | 0.04 ± 0.25 | 0.499 |
| Days of stay (resp hosp) | 0.40 ± 2.15 | 0.22 ± 1.4 | 0.321 |

P-Value (Chi-squared or T-student). **Bolded** text highlights variables with statistically significant differences (p≤0.05).

COPD: chronic obstructive pulmonary disease; ACO: asthma-COPD overlap; SABA: short-acting beta agonists LABA: long-acting beta agonists; LAMA: long-acting muscarinic antagonists; ICS: inhaled corticosteroids; OCS: oral corticosteroids (at least one prescription during the study period); FEV1: forced expiratory volume in 1^st^ second; FVC: forced vital capacity; postBD: post-bronchodilator; BDR: bronchodilator response; Eos: eosinophils; ED: emergency department; Hosp: hospitalization; Resp hosp: respiratory hospitalization; No: number.
