# Supplementary material for: ACO: Time to move from the description of different phenotypes to the treatable traits
Source: PLoS One. 2019 Jan 24;14(1):e0210915. doi: 10.1371/journal.pone.0210915 (PMC6345463; doi:10.1371/journal.pone.0210915)
Supplement: S2 Table — P-Value (Chi-squared or T-student). Bolded text highlights variables with statistically significant differences (p≤0.05). vCOPD: chronic obstructive pulmonary disease; ACO: asthma-COPD overlap; SABA: short-acting beta agonists LABA: long-acting beta agonists; LAMA: long-acting muscarinic antagonists; ICS: inhaled corticosteroids; OCS: oral corticosteroids (at least one prescription during the study period); FEV1: forced expiratory volume in 1st second; FVC: forced vital capacity; postBD: post-bronchodilator; BDR: bronchodilator response; Eos: eosinophils; ED: emergency department; Hosp: hospitalization; Resp hosp: respiratory hospitalization; No: number. (DOCX) [file pone.0210915.s002.docx]

**Table S2.** Demographic and clinical characteristics of COPD (no-ACO) and smoking asthmatic (SA) populations.

|  | **COPD (n=438)** | **SA (n=83)** | **P-Value** |
| --- | --- | --- | --- |
| Male | 349 (79.7%) | 47 (56.6%) | **<0.001** |
| Age, years | 67.66 ± 9.12 | 60.98 ± 9.67 | **<0.001** |
| Pack years | 16.12 ± 18.89 | 20.29 ± 23.61 | 0.078 |
| **Comorbidities** |  |  |  |
| Atrial fibrillation | 87 (19.9%) | 9 (10.8%) | 0.052 |
| Anxiety, No. (%) | 131 (29.9%) | 35 (42.2%) | **0.028** |
| Osteoporosis, No. (%) | 49 (11.2%) | 17 (20.5%) | **0.020** |
| Allergic rhinitis, No. (%) | 30 (6.8%) | 16 (19.3%) | **<0.001** |
| GERD, No. (%) | 34 (7.8%) | 12 (14.5%) | **0.049** |
| Nasal polyps, No. (%) | 2 (0.5%) | 3 (3.6%) | **0.007** |
| **Treatment** |  |  |  |
| SABA | 195 (44.5%) | 59 (71.1%) | **<0.001** |
| LAMA | 318 (72.6%) | 48 (57.8%) | **0.007** |
| LAMA-LABA | 62 (14.2%) | 4 (4.8%) | **0.019** |
| ICS | 21 (4.8%) | 7 (8.4%) | 0.178 |
| LABA-ICS | 232 (53.0%) | 71 (85.5%) | **<0.001** |
| OCS | 156 (35.6%) | 35 (42.2%) | 0.256 |
| **Lung function** |  |  |  |
| FVC postBD, liters | 3.16 ± 0.91 | 3.21 ± 0.80 | 0.643 |
| FVC postBD, % reference | 85.50 ± 18.24 | 87.44 ± 14.52 | 0.288 |
| FEV1 postBD, liters | 1.65 ± 0.64 | 1.71 ± 0.59 | 0.422 |
| FEV1 postBD, % reference | 58.91 ± 19.34 | 60.63 ± 17.96 | 0.431 |
| FEV1/FVC postBD | 52.11 ± 12.70 | 53.08 ± 12.80 | 0.526 |
| BDR |  |  | **<0.001** |
| - Negative | 370 (84.5%) | 54 (65.1%) | **<0.001** |
| - Positive (≥200ml and ≥12%) | 68 (15.5%) | 22 (26.5%) | **<0.001** |
| - Highly-positive (≥400ml and ≥15%) | 0 (0%) | 7 (8.4%) | **<0.001** |
| **Eosinophils count** |  |  |  |
| Mean Eos | 0.15 ± 0.07 | 0.23 ± 0.22 | **<0.001** |
| Median Eos | 0.14 ± 0.08 | 0.22 ± 0.22 | **<0.001** |
| Maximum Eos | 0.27 ± 0.19 | 0.33 ± 0.26 | **0.025** |
| **Use of health services** |  |  |  |
| ED visits | 1.74 ± 2.08 | 1.78 ± 2.29 | 0.886 |
| Hosp all cause no. | 1.14 ± 1.50 | 1.18 ± 1.89 | 0.859 |
| Days of stay (all cause hosp) | 9.60 ± 18.38 | 10.33 ± 25.92 | 0.808 |
| Resp hosp no. | 0.06 ± 0.28 | 0.17 ± 0.54 | **0.005** |
| Days of stay (resp hosp) | 0.40 ± 2.15 | 1.24 ± 4.77 | **0.011** |

P-Value (Chi-squared or T-student). **Bolded** text highlights variables with statistically significant differences (p≤0.05).

COPD: chronic obstructive pulmonary disease; ACO: asthma-COPD overlap; SABA: short-acting beta agonists LABA: long-acting beta agonists; LAMA: long-acting muscarinic antagonists; ICS: inhaled corticosteroids; OCS: oral corticosteroids (at least one prescription during the study period); FEV1: forced expiratory volume in 1^st^ second; FVC: forced vital capacity; postBD: post-bronchodilator; BDR: bronchodilator response; Eos: eosinophils; ED: emergency department; Hosp: hospitalization; Resp hosp: respiratory hospitalization; No: number.
